# Prevalence and characteristics of thoracic diffuse idiopathic skeletal hyperostosis in 3299 black patients

**DOI:** 10.1038/s41598-021-01092-x

**Published:** 2021-11-12

**Authors:** Hiroyuki Yoshihara, Vidushan Nadarajah, Evan Horowitz

**Affiliations:** Department of Orthopaedic Surgery and Rehabilitation Medicine, SUNY Downstate Medical Center, 450 Clarkson Ave, Brooklyn, NY 11203 USA

**Keywords:** Spondyloarthritis, Bone, Epidemiology

## Abstract

The purpose of this study was to examine the prevalence and characteristics of thoracic diffuse idiopathic skeletal hyperostosis (T-DISH) in the Black patients using the computed tomography (CT) analysis. This study is a cross-sectional study. All patients who underwent chest CT for the trauma screening and whose race was categorized as “Black” on the questionnaire were recruited in the study from Mar 2019 to Mar 2020. Demographic data, including age, sex, body mass index (BMI), and presence of diabetes mellitus (DM), were recorded. A total of 3299 Black patients (1507 women and 1792 men) were included for the analysis. The prevalence of T-DISH was 7.7% (255 patients), with 8.6% for females and 7.0% for males. The highest prevalence was observed in patients at the age of 70 years (11.7%), followed by the age of 80 years (10.5%). The highest prevalence level of T-DISH segment was at T8, followed by T9, and T7. The most frequent number of contiguous vertebrae was seven (21%). BMI was not associated with T-DISH. The presence of DM was significantly higher in male patients with T-DISH than those without T-DISH (P = 0.02).

## Introduction

Diffuse idiopathic skeletal hyperostosis (DISH) is a skeletal disease categorized by progressive ossification of the anterior longitudinal ligament and bone proliferation at the enthesis^1,2^. The pathophysiology is not fully understood. The criteria established by Resnick and Niwayama^2^ in 1976 is most commonly used for the diagnosis of DISH: (1) The presence of calcification and ossification along the ventrolateral aspects of at least four contiguous vertebral bodies; (2) The preservation of disc spaces; (3) The absence of apophyseal joint degeneration or sacroiliac inflammatory changes. Although DISH is assumed to be an asymptomatic condition in most affected individuals, several clinical symptoms have been reported including pain, dysphagia at cervical level, restriction of spinal movements, and increased risk of unstable spinal fractures after trauma^3,4^.

Previous epidemiological studies have investigated the prevalence of DISH, which is reported to be 2.6–19.8%^5–16^. The evaluation was traditionally conducted by the plain chest or abdominal radiographs. Recently, computed tomography (CT) scans are used for the evaluation of DISH. Then, the diagnosis using CT scan images has been reported to be more reliable than that by conventional radiographs^12^. However, the prevalence of DISH using CT scans was reported only in the Asian populations, and there is no study that reported the prevalence of thoracic DISH (T-DISH) in the Black population using the CT analysis.

The purpose of this study was to examine the prevalence of T-DISH in the Black patients within the United States who underwent multi-slice chest CT for the trauma screening. We also analyzed the characteristics of the T-DISH in the Black patients. Furthermore, previous studies reported that high body mass index (BMI) and the presence of diabetes mellitus (DM) are the risk factors for DISH^13,14,17^; thus, the association of T-DISH with BMI and DM was examined.

## Material and methods

### Study participants

This study was approved by our institutional review board (Kings County Hospital Center, STUDY00002324). A cross-sectional review of a prospectively registered database in New York City Health and Hospitals (11 hospitals in New York City) was performed. All methods were carried out in accordance with relevant guidelines and regulations. Informed consent was obtained from all subjects or, if subjects are under 18, from a parent and/or legal guardian. All patients who underwent chest CT for the trauma screening and whose race was classified as “Black” on the questionnaire were recruited in the study from Mar 2019 to Mar 2020. We used “Black” because this was the term used on the questionnaire and “Black” can include “African-American” and “Caribbean-American.” The patients with previous thoracic spine surgery were excluded from the study. Demographic data, including age, sex, BMI, and presence of DM were recorded. These data were based on a questionnaire completed at the initial visit to the trauma center.

### Radiographic assessment

The definitive diagnosis of T-DISH was made according to the modified Resnick and Niwayama criteria based on the CT scan images, proposed by Oudkerk et al.^17^; (I) Sagittal viewing plane is used to diagnose DISH. (II) The scan is viewed in a fixed window level of W/L 800/2000. (III) The outer contour of the flowing ossifications intersects the vertebral body at > 90° respecting the globally flowing character of the bridging ossification. (IV) Severe disc degeneration is excluded from the diagnosis of DISH. (V) A minimum of three contiguous intervertebral levels or four contiguous vertebrae with connecting flowing ossifications is necessary (Table 1). The level involved by T-DISH was evaluated using sagittal reconstruction images.

**Table 1:** Modified Resnick and Niwayama Criteria for Diagnosis of DISH based on CT by Oudkerk et al.^17^.

I. At least four contiguous vertebrae or alternatively three contiguous disc level are bridged
II. Fixed (bone) settings to prevent false positive and false negative cases
III.The angle formed by an osteophyte in relation to vertebral bodies should be larger than 90°
IV. In cases of mild or moderate degenerative disc changes in combination with flowing ossifications, the diagnosis of DISH can be established

*DISH* diffuse idiopathic skeletal hyperostosis, *CT* computed tomography.

All CT scans were acquired using a multi-detector CT with the following parameters: slice thickness, 1.25 mm; pixel size, 0.352 mm; tube rotation speed, 0.5 s; tube current, 200 mA; beam collimation, 40 mm; beam pitch, 0.9; and voltage, 120 kV. Reconstructed sagittal images were analyzed with a Picture Archiving and Communication System (PACS) viewer.

Images were reviewed by two orthopaedic surgery residents (V.N. and E.H.), who were trained by an experienced orthopaedic spine surgeon (H.Y.) to identify and classify DISH via CT scan. Before reviewing images, the testers (V.N. and E.H.) read the same images of 40 patients to verify inter- and intra- observer agreement. Intra-observer agreement was determined with evaluation of the same 40 patients at separate time points one month apart.

### Statistical analysis

Student *t* test and χ2 test were used when appropriate. A *P* value of less than 0.05 was considered statistically significant. Statistical analysis was performed using JMP Pro, Version 15 software (JMP®, Version 15*.* SAS Institute, Cary, NC).

## Results

### Demographic data

A total of 3299 Black patients were included in the analysis. Of the 3299 patients, 1507 were females and 1792 were males, with the mean age of 57.6 ± 17.1 years (range, 5–103) (Table 2). BMI data were available for 92.2% of the patients. Mean BMI was 27.8 ± 7.0 kg/m. Based on the questionnaire, 1121 patients (34.0%) had the diagnosis of DM.

**Table 2:** Distribution of study population according to T-DISH ( +) or ( −).

	T-DISH ( +)	T-DISH ( −)	ALL	P value
**Sex**	**N**	**N**	**N (%)**	
Male	126	1666	1792 (54.3)	0.1
Female	129	1378	1507 (45.7)	
Male to Female	1.0:1.0	1.2:1	1.2:1	
**Age, mean**	62.1 (± 15.5)	57.2 (± 17.2)	57.6 (± 17.1)	** < .0001**
**Age group, yrs**	**N (%)**	**N (%)**	**N**	
< 10	0 (0)	7 (100)	7	
10–19	1 (3.2)	30 (96.8)	31	
20–29	12 (5.5)	208 (94.6)	220	
30–39	12 (3.8)	307 (96.2)	319	
40–49	16 (4.8)	321 (95.3)	337	
50–59	55 (7.7)	660 (92.3)	715	
60–69	68 (8.1)	772 (91.9)	840	
70–79	64 (11.7)	485 (88.3)	549	
80–89	24 (10.5)	204 (89.5)	228	
90–99	3 (5.9)	48 (94.1)	51	
≥ 100	0 (0)	2 (100)	2	
Total	255	3044	3299	
**BMI**, kg/m^2^	27.5 (± 6.8)	27.8 (± 7.0)	27.8 (± 7.0)	0.76
Male	27.3 (± 6.7)	28 (± 7.0)	28 (± 7.0)	0.37
Female	27.6 (± 6.9)	27.5 (± 6.9)	27.5 (± 7.0)	0.60
**Diabetes (DM)**				
DM +	94	1027	1121	0.34
DM −	161	2017	2178	
Male DM +	60	617	677	**0.02**
Male DM −	66	1049	1115	
Female DM +	34	410	444	0.48
Female DM −	95	968	1063	

*T-DISH* thoracic diffuse idiopathic skeletal hyperostosis; *N* number, *BMI* Body mass index.

### Prevalence and characteristics of T-DISH

**Figure 1:**
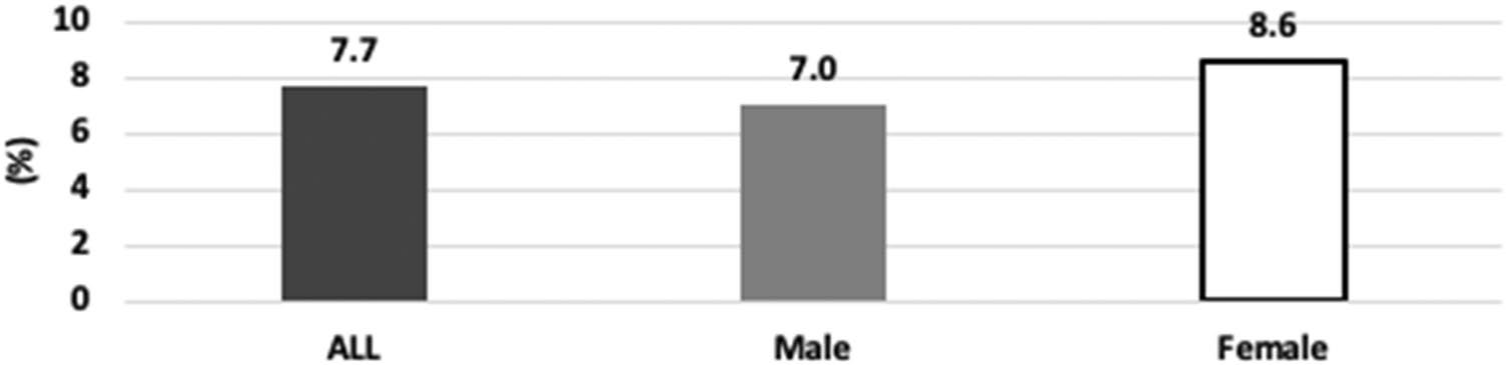
Prevalence of thoracic diffuse idiopathic skeletal hyperostosis.

The kappa coefficient of inter- and intra-observer agreements were 0.90 and 0.91 (V.N. 0.87, E.H. 0.95), respectively. Of the 3299 patients, there were 255 patients with T-DISH (129 females and 126 males) (Table 2). The overall prevalence of T-DISH was 7.7%, with 8.6% for females and 7.0% for males. (Fig. 1). No difference was found in the prevalence of T-DISH between female and male patients (8.6% vs 7.0%, *P* = 0.1). The mean age of patients with T-DISH was significantly higher than that without T-DISH (62.1 vs 57.2, *P* < 0.001).

**Figure 2:**
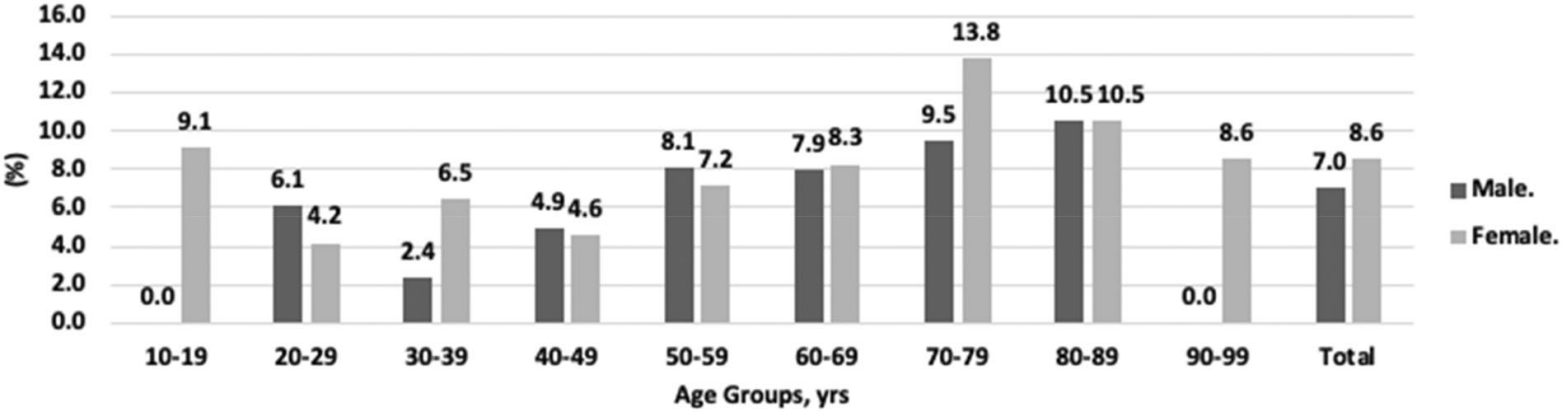
Age distribution of the prevalence of thoracic diffuse idiopathic skeletal hyperostosis.

**Figure 3:**
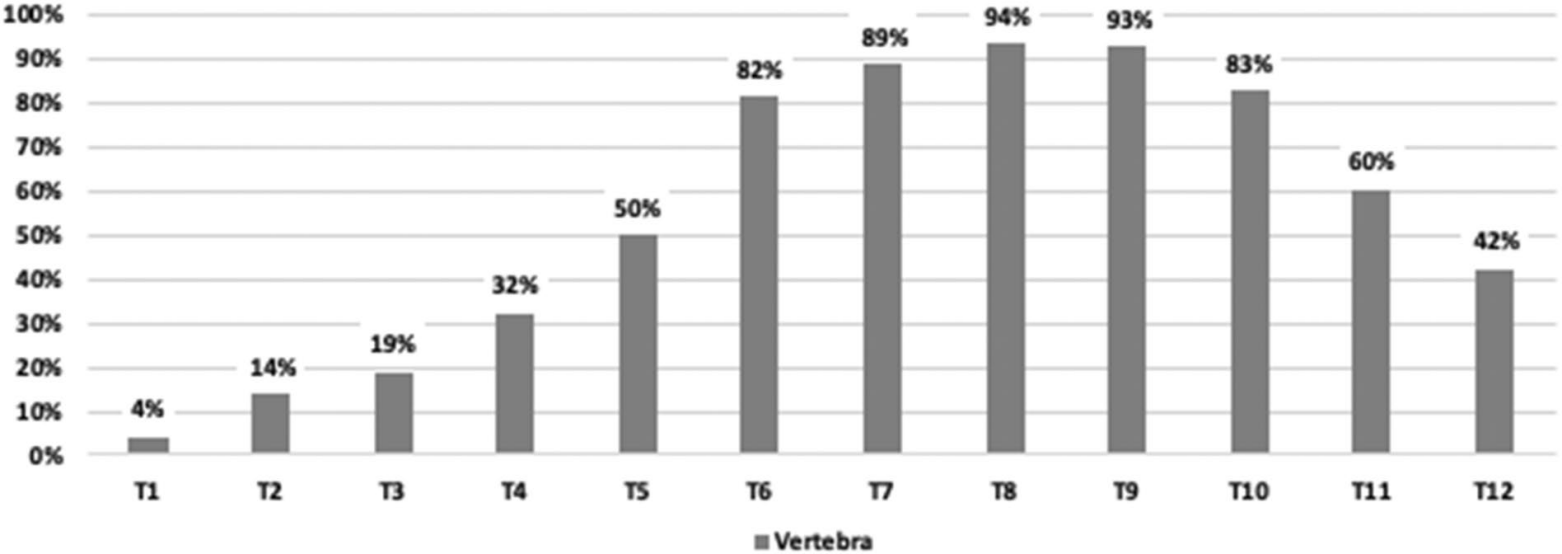
Distribution of the thoracic diffuse idiopathic skeletal hyperostosis based on the spinal segment.

**Figure 4:**
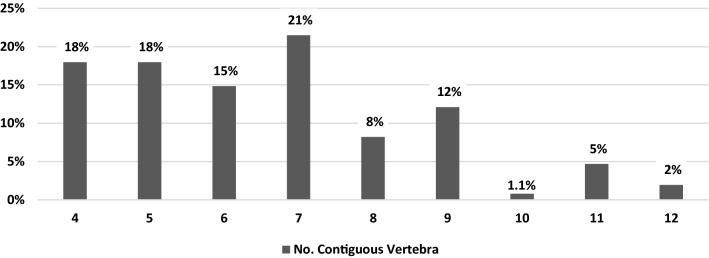
Number of the contiguous vertebrae of the thoracic diffuse idiopathic skeletal hyperostosis.

With regard to the prevalence of T-DISH among each 10-year age group, the highest prevalence was observed in patients at the age of 70 years (11.7%), followed by the age of 80 years (10.5%), and the age of 60 years (8.1%) (Fig. 2). The distribution of T-DISH in the thoracic segments is shown in Fig. 3. The highest prevalence level of T-DISH segment was at T8 (94%), followed by T9 (93%), and T7 (89%). The number of contiguous vertebrae of T-DISH is shown in Fig. 4. The most frequent number of contiguous vertebrae was seven (21%), followed by four (18%), and five (18%).

### Association between T-DISH and BMI/DM

There was no difference in the mean BMI between patients with and without T-DISH (27.5 vs 27.8, *P* = 0.76) (Table 2). No difference was found in the mean BMI between female patients with and without T-DISH (27.6 vs 27.5, *P* = 0.60), and between male patients with and without T-DISH (27.3 vs 28.0, *P* = 0.37). There was no difference in the presence of DM between patients with and without T-DISH (36.9% vs 33.7%, *P* = 0.34) (Table 2). No difference was found in the the presence of DM between female patients with and without T-DISH (26.4% vs 29.8%, *P* = 0.48). The presence of DM was significantly higher in male patients with T-DISH than those without T-DISH (47.6% vs 37.0%, *P* = 0.02).

## Discussion

Our study revealed that the prevalence of T-DISH in the Black patients was 7.7%, who underwent chest CT for the trauma screening. The prevalence of T-DISH has been reported to be 2.6–17%^5–7,9,10,14^ (Table 3). However, it is difficult to compare with the prevalence of T-DISH in the previous epidemiological literature due to the differences in the diagnostic modality, diagnosis criteria, and the study population.

**Table 3:** Previous studies reporting the prevalence of T-DISH.

Authors (year)	Race	Sample size	Modality	Prevalence
Julkunen et al. (1975)	White	8993	Chest x-ray	2.6% (M 3.8%, F 2.6%)
Cassim et al. (1990)	African	1500	Chest x-ray	3.9% (M 3.8%, F 4.2%)
Weinfeld et al. (1997)	Mixed race	2364	Chest x-ray	NA (M 25%, F 15%)
Kim et al. (2004)	Asian	3595	Chest x-ray	2.9% (M 5.4%, F 0.8%)
Westerveld et al. (2008)	White	501	Chest x-ray	17% (M 22.7%, F 12.1%)
Mori et al. (2017)	Asian	3013	Chest CT	8.7% (M 13%, F 2.5%)

*T-DISH* thoracic diffuse idiopathic skeletal hyperostosis; *CT* computed tomography, *M* male; *F* female.

Traditionally, the evaluation of DISH was conducted by the plain chest or abdominal radiographs. However, Hirasawa et al.^12^ implied that it seems too ambiguous to judge the presence of spinal column continuity only based on the radiographs and rely on the reliability and accuracy as an interpretation. They compared the prevalence of DISH assessed by reconstructed CT of the chest to pelvis with that evaluated by plain radiographs of the chest and abdomen. The results revealed that the prevalence of DISH based on CT was 27.1% and that based on radiographs was 17.6%^12^. This fact confirms that CT scan is a better diagnostic modality for the evaluation of DISH.

We evaluated T-DISH using the modified Resnick and Niwayama criteria, instead of Resnick and Niwayama criteria. Oudkerk et al.^17^ proposed the modified Resnick and Niwayama criteria for the diagnosis of DISH on chest CT (Table 1). They evaluated inter-observer agreement related to the diagnosis of DISH on chest CT and found inter-observer agreement was fair or poor by kappa analysis using the Resnick and Niwayana criteria^17^. This indicates that the reliability of the original criteria on chest CT may be disputable. In contrast, they also evaluated the modified Resnick and Niwayama criteria and demonstrated that the modified criteria to identify DISH on CT scan led to moderate to excellent agreement between the observers with different levels of experience and expertise^17^. In our study, the results of kappa coefficient of intra- and inter-observer agreement also demonstrated a substantial and excellent agreement (0.90 and 0.91). Thus, the modified Resnick and Niwayama criteria is more reasonable than the original criteria to evaluate DISH on CT scan images.

The range of age in the study population also affects the prevalence of DISH. Most previous reports only included people who are older than 40 years and the average age is around 65 years^6,7,9,10,12^. In contrast, our study includes the range of age from 5 to 105 and the average age is 57.6 years. We believe our study population reflect more accurate prevalence of T-DISH compared with the previous reports which only included people older than 40 years.

The prevalence of previous reports showed a considerable difference by race. Weinfeld et al.^7^ showed that the prevalence was different between ethnic groups. They reported that DISH is less common in the Black, Native-American and Asian populations, although the sample size of their study was small^7^. Our study revealed the prevalence of T-DISH in the Black population seems to be not as low based on the CT analysis. Regarding the prevalence of T-DISH in the Black population, Cassim et al.^6^ reported it was 3.9% in 1500 African in South Africa, using conventional chest radiographs; however, there is no study that reported the prevalence of T-DISH in the Black population using the CT scan. Our study first reported the prevalence of T-DISH in the Black population using the CT scan and modified Resnick and Niwayama criteria.

This study demonstrated that the prevalence of T-DISH was 8.6% in females and 7.0% in males. This is an interesting finding because most of the previous studies showed the prevalence of DISH was higher in male patients compared with female patients. One study showed the prevalence of DISH was higher in female patients. Cassim et al.^6^ found the prevalence of T-DISH in the African population was 4.2% in females and 3.9% in males. These results may suggest that female dominance in the prevalence of T-DISH is specific to the Black population.

The prevalence of T-DISH was the highest at the age of 70 years (11.7%), followed by the age of 80 years (10.5%). This is consistent with other previous reports^12,14,15^. Mori et al.^14^ found that T-DISH was observed after the age of 40 years and the prevalence of T-DISH rose rapidly with age until its peak distribution, the age of 70 years. Interestingly, in our study, the prevalence of T-DISH below the age of 40 years was 4.3% (25/577). Recent studies that included all age groups and analyzed CT or PETCT revealed there was no or minimum DISH patients below the age of 40 years in Japanese^14,15^. We do not have a clear explanation for this. Patients with genetic diseases resulting in abnormalities of the parathyroid hormone-calcium-phosphate pathway may develop ossification of spinal ligaments and/or ectopic calcifications in young individuals^18^, although we were unable to obtain such information of those patients. Or this finding may be also specific to the Black population.

In our study, the highest prevalence level of T-DISH segment was at T8, followed by T9, and T7. Hirasawa et al.^12^ reported that T-DISH most commonly occurred at T7/8, T8/9 and T9/10. Hiyama et al.^15^ performed a whole-spine CT study and found that most of the ossification occurred in the middle and lower thoracic spine: T8 (88%), T9 (91%), and T10 (85%). They described that such result may suggest that an anatomical effect which these vertebrae are vulnerable to compressive mechanical stress because T8 is located almost at the peak of the physiological spinal kyphosis^15^. The most frequent number of contiguous vertebrae was seven (21%), followed by four (18%), and five (18%). In Mori’s study, the most frequent number of contiguous vertebrae was eight in Japanese^14^.

Previous studies reported that high BMI and the presence of DM are the risk factors for DISH^13,14,19^. In Japanese patients, Fujimori et al.^13^ reported that high BMI was associated with DISH in female subjects; in contrast, Mori et al.^14^ found a significant association between T-DISH and high BMI in male subjects. However, our study showed no association between T-DISH and BMI in the Black patients. A proposed mechanism of DM for DISH is the prolonged and high levels of insulin or insulin-like growth factors occurring in DM patients, stimulating new bone growth^19^. Our study revealed that the presence of DM was significantly higher in male patients with T-DISH than those without T-DISH (*P* = 0.02). Similarly, Fujimori et al.^13^ found that high glucose level was associated with DISH in Japanese males. However, our study showed no association between T-DISH and DM in female patients.

Our study is limited by several limitations. First, this study population may not represent the general population and may have a selection bias due to the limited area (New York City). However, as it is unethical to take CT scans of normal volunteers, we consider that our study participants may represent the best possible sampling for the general population. Second, we could not evaluate the whole spine or sacroiliac joint. Third, we were unable to differentiate Type 1 and Type 2 DM. Type 2 DM has been reported to be associated with DISH, whereas Type 1 DM is not^20^. Fourth, we were unable to obtain genetic disease information such as abnormalities of the parathyroid hormone-calcium-phosphate pathway, particularly in young patients. Previous studies reported the correlation between DISH and abnormalities of that pathway^21,22^. Fifth, the study lacks sufficient metabolic data, including both anthropometric (waist circumference for metabolic syndrome) and chemical blood measurements. Despite these limitations, we believe that our data are invaluable because this was the first study to examine the prevalence of T-DISH using CT analysis in the Black population.

## Conclusion

The prevalence of T-DISH was 7.7% in the 3299 Black patients who underwent chest CT for the trauma screening, with 8.6% for females and 7.0% for males. The highest prevalence was observed in patients at the age of 70 years (11.7%), followed by the age of 80 years (10.5%). The highest prevalence level of T-DISH segment was at T8, followed by T9, and T7. The most frequent number of contiguous vertebrae was seven (21%). BMI was not associated with T-DISH. The presence of DM was significantly higher in male patients with T-DISH than those without T-DISH (*P* = 0.02).
